# Cat Colony Caretakers' Perceptions of Support and Opposition to TNR

**DOI:** 10.3389/fvets.2019.00057

**Published:** 2019-03-04

**Authors:** Jacquie Rand, Andrea Hayward, Kuan Tan

**Affiliations:** ^1^School of Veterinary Science, The University of Queensland, Gatton, QLD, Australia; ^2^Australian Pet Welfare Foundation, Kenmore, QLD, Australia

**Keywords:** trap, neuter, return, community, cats, management, support, opposition

## Abstract

Trap, neuter and return (TNR) is a non-lethal approach to urban cat management used effectively internationally to decrease urban cat numbers, but deemed illegal in Australia. We investigated perceived support and opposition to TNR experienced by respondents involved in TNR activities, as individuals or through organizations. TNR was initiated to reduce cat numbers, as a humane way to manage community cats, and to improve cat welfare. Many respondents sought permission from local authorities, and all received verbal permission. Perceived attitudes of stakeholders, for example authorities and neighbors, were polarized, with some supporting it and others antagonistic and threatening legal action. Respondents generally managed the colony themselves or with assistance from friends or family, and half obtained aid from a cat welfare agency. Some respondents received cash or food from stakeholders, subsidies for desexing and education on trapping. Complaints were most common from neighbors, and less from those working and living nearby the colony. Resolution was attempted with varying success, by face-to-meetings with complainants, educational flyers, cat deterrents, or relocating cats. Supportive stakeholders had similar motives to the respondents for supporting TNR, namely to reduce cat populations and improve cat welfare. These findings are important because they demonstrate the difficulty faced by individuals and organizations undertaking TNR in Australia. Given the reported effectiveness of well-managed TNR programs, and the lack of other acceptable methods for managing urban stray cats at a city level, it is recommended that TNR be legalized in Australia in urban and periurban areas to facilitate its implementation.

## Introduction

The majority of Australia's population live in urban areas where the stray cat population is estimated to range from 60 to 100 cats per 1,000 residents, which equates to between 1.2 and 2 million urban stray cats (1, 2). Urban stray cats account for ~50–70% of Australian RSPCA shelter intake of cats (3–5) and 80–90% of intake into local government animal facilities (council pounds) (2). Based on current data for Australia, it is estimated that ~3–5% of the urban stray cat population is killed in shelters and council pounds annually (2). This low level *ad hoc* culling is unlikely to reduce cat numbers in the medium to long-term, because of the prolific reproductive capacity of cats, and because culling results in increased juvenile survival and immigration of other cats into the‘area (6, 7).

Australian legislation divides cat populations as either domestic (owned) or non-domestic (feral). Depending on the state, urban stray cats with no defined owner are either classified as “non-domestic” and subject to legislation relating to feral cats, or if classified as “domestic,” are subject to animal welfare legislation relating to animal abandonment (8). Feral cats are generally located in rural or forested areas and do not depend on humans for shelter or food (9), whereas urban stray cats commonly live in close proximity to humans, and are provided with food and shelter by humans, either intentionally or unintentionally (2).

Trap, neuter and return (TNR) of urban stray cats involves their humane capture, desexing and return to location (10, 11). Typically, it also involves removing kittens and friendly adults for rehoming. Colonies managed with TNR decrease in size over time, when a high proportion are desexed and immigrant cats are rapidly removed or desexed (12–16). Effective population reduction programs have been reported in various international sites and in Australia (2, 8, 17–22). In TNR programs, cat colonies are usually provided with food, veterinary care as needed, and frequently shelter (23). Despite acknowledgment of the problems arising from urban stray cat populations, including nuisance behaviors such as fighting and soiling, concern about disease transmission to humans, pets and wildlife, and predation of native wildlife (10, 11), there is no current consensus of how the community and governments should manage urban stray cats in Australia. Cats in shelters and pounds which are in excess of those that can be rehomed, or are too poorly socialized to be rehomed, are euthanized. However, this lethal control is not wholly supported by the community, and has been demonstrated to impair the mental health of those tasked to kill them (21, 24–28).

Management of urban stray cats varies in Australia depending on state legislation, local government bylaws and landholders (8). Legally, returning unowned cats after neutering to where they were found is generally considered an offense in Australia under either domestic animal welfare legislation relating to abandonment of cats, or biosecurity and land management legislation relating to cats as pest species. An attempt to legalize TNR in NSW was made through the Animal Welfare (Population Control Programs) Bill, but it did not progress past the first reading and has since lapsed (29). To the authors' knowledge, there has been no prosecutions for participating in TNR activities to date, however, prosecutions for feeding urban strays have occurred (30). Queensland has the most restrictive legislation and only owned cats are considered domestic, and it is illegal to feed, remove (for adoption) or release “non-domestic” cats which includes urban strays, because they are considered “restricted matter” (31, 32). In most states and territories of Australia, the RSPCA is the authority legally responsible for investigating animal cruelty, for example, abandonment of pets.

In Australia, TNR is often undertaken covertly because of the threat of prosecution, and the absence of widespread support or advocacy from traditional animal welfare stakeholders. The two largest animal welfare advocacy groups are the Royal Society for the Prevention of Cruelty to Animals (RSPCA), the major not for profit organization in Australia dedicated to prevention of cruelty in animals, and the Australian Veterinary Association (AVA). At the time of writing, the AVA's official policy is “trap, neuter and return strategies have not been shown to be effective under Australian conditions as the cats often do not have a good level of welfare once released, continue to hunt and predate, and can be a significant public nuisance” (33). These findings are based on literature published between 2009 and 2011, and differ from more recent evidence of the positive effect of TNR programs, including improved health of cats, decreased stray cat numbers, and reduced cat-related complaints (2, 13–16, 21, 34). The RSPCA in their 2018 report Identifying Best Practice Domestic Animal Management in Australia suggests that poor implementation is likely to have contributed to poor outcomes in TNR programs and further research is recommended into trap-desex-adopt-or return and support programs under Australian conditions (35).

Welfare of the cats and wildlife predation are often raised as impediments to supporting TNR (36). These concerns are not supported by current research as evidenced by improved body condition of cats in TNR programs, and reduced cat numbers, therefore reducing opportunities for wildlife predation (2, 13–15, 21, 34). Despite the illegality of TNR in many jurisdictions in Australia, TNR is being practiced by citizens concerned for the welfare of stray cats. We recently published a survey of 53 respondents managing cat colonies through TNR (2). Respondents were located in all major capital cities in Australia. We documented in an Australian context the success of TNR in reducing cat numbers. For example, median colony size decreased by 31% over a median of 2 years from 12 to 7 cats, and the total number of cats decreased from 515 to 344 over 2.4 years (2).

The aims of this current study were to identify colony carers' perceptions of support and opposition to TNR from various stakeholders. Perceived challenges, at commencement and currently, faced by respondents who were involved in TNR as individuals or part of an organizations are reported. This information is important to inform whether there is a need for legislative change to facilitate TNR activities in urban and periurban areas of Australia. We second aim was to determine the reasons TNR was initiated for that colony and the perceived reasons it was supported by stakeholders.

## Materials and Methods

### Survey Design

We designed a questionnaire for individuals involved with TNR to determine their perceived experience using TNR, and specifically, their perceptions of support and opposition from various stakeholders. Because there were no published and validated survey tools incorporating the specific areas of interest for this study, the survey was developed and peer reviewed by Australian and international experts in TNR and in survey design (see Acknowledgments). We also piloted the draft questionnaire amongst some individuals involved in TNR, and the questionnaire was modified based on suggestions for change. These responses were not included in the data set.

A retrospective cross-sectional study of adult respondents involved in TNR in Australia was conducted using a convenience sample, including snowball sampling. Snowball sampling enables hard-to-reach populations to be contacted via social networks, by linking participants through a referral chain. Although snowball sampling does have limitations, including potential for bias, it manages to engage participants who are hidden and difficult to contact (37, 38). It was selected for this study as it ensures the anonymity of participants who did not wish to have their potentially illegal activities exposed. The study was approved by Bellberry Human Ethics Committee EC00450.

The survey was created using online survey software (SurveyMonkey®) and an identical downloadable Microsoft Word version was provided for anonymous postal responses for respondents concerned about traceability of IP addresses. We have previously reported data on information of TNR sites and colonies, demographics of respondents, motivations for involvement, TNR operations including feeding, trapping and desexing, identification, provision of healthcare and rehoming, funding and costs (2). In the current study, we report responses from 30 respondents who answered one or more questions on stakeholder support. These respondents represented a subset of the original cohort of 53 respondents and an additional four respondents to the questionnaire.

### Data Collection

A link to the questionnaire were hosted on a website for a not for profit, companion animal, re-homing organization (Maggie's Rescue). A downloadable version of the questionnaire was also available on the website for respondents to send back by mail if preferred. Emails advertising the survey were sent to the contact list for Maggie's Rescue (100 contacts). Respondents were requested to complete the survey if they were involved with TNR, and to forward it on to others they knew who were involved in TNR, utilizing a “snowballing” effect. To maximize response rate, a modified form of Dillman's Tailored Method (39) was utilized with 2 email reminders sent at ~1 week intervals. No inducements were offered to participants for participation in the survey.

Respondents were advised that that TNR could be considered an offense in some jurisdictions and were permitted to withdraw from the survey, or alternatively to complete the survey as a Word document and submit anonymously by post to Maggie's Rescue. The study focused on TNR in urban areas involving stray cats. Respondents were instructed to only complete the questionnaire if TNR was conducted in urban areas, and not in bushland, National or State parks or reserves. A total of 57 responses were received between September and January 2017, with ~50% of respondents completing the entire survey, which took ~1 h.

In this current study, 30 of the 57 respondents engaged in TNR in Australia who completed the questionnaire on their activities, answered one or more questions regarding stakeholder support. Respondents were asked about their experiences with colonies they or their organization was using TNR to manage in urban areas of Australia. They were instructed that the questionnaire related to community cats managed by TNR, and not to colonies where desexing did not occur. Respondents were involved with TNR as individuals or as part of an organization. Specifically respondents were asked questions relating to perceived support or opposition to TNR from a variety of stakeholders. Stakeholders were defined as a person, group or organization that had an active interest in the TNR activity. Potential stakeholders included authorities responsible for compliance with legislation related to pest species (state government, local government) and animal welfare (RSPCA in most states), and police. Stakeholders also included landholders, business owners, neighbors, workers and residents where the colony was located, administrators of schools, hospitals, universities, and public or private housing, veterinarians and welfare agencies.

Numbers of respondents to each question are indicated where relevant. Descriptive statistics are only reported because of the small sample size. Although the study was not designed as mixed methods research, comments from participants were included to enrich data from the limited response rate.

## Results

Of the 30 respondents who were engaged in TNR activities in Australia and answered one or more questions on attitudes of stakeholders, 28 (93%) were female and most (39%) were aged 46–55 (median age group bracket = 46–55). Respondents were involved with managing colonies located in NSW (15/28), Victoria (6/28,) Queensland (5/28), Western Australia (4/28), and (ACT (1/28) (one respondent managed colonies in NSW and ACT). Most colonies were at private residential homes (29%) and alleyways (13%) or industrial areas (13%) (Table 1). More (71%, 20/28) respondents conducted TNR as an individual than as part of an organization (34%, 10/28).

**Table 1:** Location of 52 colonies where respondents (*n* = 28) were conducting TNR.

**Location for the colony/ies where 28 respondents were conducting TNR**	**Number and proportion of colonies at each location (*n* = 52)**
Private residential home	15 (29%)
Alleyway or street	7 (13%)
Industrial area or factory complex	7 (13%)
Other: beside railway line, shopping center, derelict hospital, derelict hoarders house, community facility	5 (10%)
Car park (around shops, fast food outlets, or municipal car park)	4 (8%)
University	4 (8%)
Government housing complex e.g., public housing.	3 (6%)
Urban park or reserve	3 (6%)
Vacant block or vacant building	2 (4%)
Hospital	1 (2%)
Private housing complex e.g., residential development; gated community	1 (2%)

### Reasons Why TNR Was Begun for That Colony

Respondents were asked why TNR was commenced at the colony, and the two most common reasons provided by the 21 respondents were that TNR was an effective way to reduce the community cat population over time (86% of respondents), and it was a humane approach to cat management (81%) (Table 2A). Other reasons were to improve the health and welfare of the cats and kittens. Nearly one third indicated that the organization they belonged to was committed to TNR.

**Table 2A:** Reasons respondents began TNR at that colony. Respondents could select more than one reason.

**Reasons TNR commenced at colony**	**Number and % of respondents selecting option (*n* = 21)**
Effective strategy to reduce the community cat population over time	18 (86%)
Because this is a humane approach to managing community cats	17 (81%)
To improve the welfare of cats/kittens	16 (76%)
To improve the health of cats/kittens	13 (62%)
The organization I belong to is committed to TNR	6 (29%)
Because of complaints to municipal authorities	2 (10%)
The organization I belong to is funded to do a TNR program	1 (5%)
Other reason/s	7 (33%)

In free form comments, some respondents cited the reason they began TNR was for humane population control and because of an inability to get assistance from cat welfare or other agencies. For others it was to manage the cats for individuals unable to care for the cats on their property, for example, elderly with dementia or hoarders (Table 2B).

**Table 2B:** Free-form comments on reasons respondents began TNR at that colony.

**HUMANE STRAY CAT CONTROL**
“Because it is the most effective, ethical and non-abusive way to manage animal populations” “A resident was feeding cats but not desexing, and this was causing a population explosion” “We just happened to find some kittens 1 day and it started from there—in the beginning there were 28!” “To control cats at a hoarder's property and for an elderly person with dementia”
**PROTECT STRAY CATS**
“The council was starting to trap and kill part of the colony. We started laying traps to save them”

### Perceived Awareness of TNR by Authorities at Commencement and Permissions Sought

Respondents were asked whether authorities were aware that TNR had commenced for this colony. Possible authorities were described as state or local government (council—equivalent to counties in USA), RSPCA, property or business owner, owner or manager of the business, or administration of school, hospital, university, government housing complex, privately owned housing complex. In most situations, permission from more than one authority was likely required, for example, state and local government. Only a minority believed authorities were aware that TNR had commenced (17%; 4/23), while most did not know if authorities were aware (43%; 10/23) or believed that the authorities were not aware (39%; 9/23).

More than half (57%; 10/18) of the respondents sought permission from one or multiple authorities, with permission sought most commonly sought from the property owners (*n* = 6). Less frequently permission was sought from the local government agency (council), (*n* = 1), university administration (*n* = 1), management of government housing (*n* = 1) or from the owner or manager of the business (*n* = 1). None sought approval from the relevant state government office associated with biosecurity or land management, or from the RSPCA (the agency in most states legally responsible for investigating animal cruelty, including abandonment).

Of those who sought permission from agencies or authorities, all (10/10) received permission, which was nearly always verbal (90%, *n* = 9). One respondent indicated that additional paperwork was required for cat registration with the municipality, and another required campus approval for a trial period. Only one of the respondents received an email or hard-copy letter stating approval was granted.

### At the Commencement of TNR, Perceptions of Various Stakeholders

#### Perceived Awareness and Attitudes of Landowners or Authorities Responsible for the Land That the Colony Was Occupying

At the commencement of TNR at the colony, 35% respondents (*n* = 20) believed that the landowners or authorities responsible for the land the colony was occupying were not aware it was occurring, while others (20%) believed they were aware but did not acknowledge TNR (Table 3). Only a minority (15%) indicated landowners supported TNR, although only one provided assistance which was in the form of funds for desexing (university management). Of those that reported negative attitudes, one respondent indicated that landowners were antagonistic, although they did not prevent TNR, another described the behavior as tense, and one respondent was threatened with legal action.

**Table 3:** At commencement of TNR, perceived awareness and attitudes of landowners or authorities responsible for land the colony was occupying.

**Reasons**	**Landholder attitudes toward the colony at commencement of TNR (% of respondents), (*n* = 20)**
Threatened to take legal action	1 (5%)
Very antagonistic but took no direct action to prevent occurrence	1 (5%)
Tension only	1 (5%)
I do not think they were aware of it occurring	7 (35%)
The authority/authorities were aware of it but did not acknowledge they were aware of it occurring	4 (20%)
The authority/authorities supported it but did not provide assistance with resources (in-kind or cash)	2 (10%)
The authority/authorities supported it and provided assistance with access to resources (e.g., human resources, desexing vouchers or assistance with desexing, traps, other assistance or cash)	1 (5%)
Don't know/ Not applicable	3 (15%)

Free-form comments tended to elaborate on the more unsupportive behaviors of landowners or authorities responsible for the land the colony was occupying. For example, “my work place advised that if I wanted to do it, it was at my cost, otherwise they would call the RSPCA to have the cats removed and destroyed,” and “they have no interest in it, I believe they would think it unnecessary.”

#### Influential Stakeholders Who Facilitated TNR Initially at the Colony

For respondents to the questionnaire, we defined an influential stakeholder as “a person, group or organization that takes an active interest in the activity, and in this case influences. These could be people in positions of authority in the animal welfare sector, such as municipal employees, councilors, shelters, also landowners, and members of the community who may have taken a leadership role.” When asked to provide free-form descriptions about who were the most influential stakeholders who initially helped the TNR work on this colony, 50% (8/16) said that involvement by a community cat welfare agency was in various ways the most helpful. Other respondents indicated friends, their partner or neighbors helped with the TNR work at the colony (44%; 7/16). One respondent was reluctant to disclose influential stakeholders, because TNR was illegal in their state. Some respondents positively commented on stakeholders' influence facilitating the continuation of TNR, with two commenting positively on support from police (Table 4).

**Table 4:** Free-form comments on positive actions toward respondents from stakeholders.

**SUPPORT RECEIVED FROM POLICE**
“We have police and security aware, and who help us, and check-in with us at night (that we are OK).” “I was aware that it is not strictly supported by ‘the authorities’. However, many times I met up with police, who I suppose thought is strange to see a woman in corporate suit and high heels lurking about in odd places after dark. As I became known, and I had several discussions, and thankfully was never ‘moved along’.” “Several police actually left cat food for me with notes a few times, as did locals.” “I spoke to anyone who wished to understand my actions, and for the most part I would get full or tacit approval.” “The police would happily wave and nod my way if ever they saw me.”
**SUPPORT RECEIVED FROM HOUSING MANAGER**
“The housing representative rang us and asked for help. Later the same person called council to intervene (because) we were making progress—we had desexed seven cats, removed seven kittens, four were very sick, then retrieved two adults taken to the pound for rehoming.”
**SUPPORT FROM LOCAL RESIDENTS**
“In two locations, locals took over the feeding and reporting duties, and also adopted a couple of cats.”
**SUPPORT FROM COMMUNITY CAT WELFARE GROUPS**
“I started my own TNR, not even knowing that this is what it was, after being given assistance from a community cat welfare agency on-line and over the phone.”

When asked to provide free-form comments on how these stakeholders influenced the commencement of TNR at this colony, many respondents were given verbal and material support. Some were educated on trapping, some stakeholders fundraised to subsidize the medical costs, and rescue groups offered subsidized desexing. Some stakeholders assisted with socialization of cats before adoption and some gave access to locations where stray cats could be trapped. One respondent commented that her over-riding strategy if someone showed interest, was to recruit them to assist or take over. “It is amazing how motivated people can become when they know they can make a difference!”

#### At Commencement, Complaints or Issues With Neighbors, People Working or Living Where the TNR Colony Was Located

Complaints were most frequently reported from neighbors, with two thirds of respondents reporting complaints or issues at commencement of TNR (Table 5), and less frequently (45%) these issues occurred with people living where the colony was located, and least frequently complaints were from people working adjacent to where the colony was located. One respondent reported a hostile resident re-trapped one cat who had been neutered and returned to the colony, then sent the cat to the council pound.

**Table 5:** When TNR commenced, were there complaints or issues with neighbors, people working or living adjacent to this colony?

**Were there complaints or issues?**	**Neighbors (*n* = 22)**	**People working adjacent (*n* = 21)**	**People living (*n* = 22)**
Yes	15 (68%)	7 (33%)	10 (45%)
No	7 (32%)	14 (67%)	12 (55%)

Although a few respondents made no attempt to resolve complaints or issues with neighbors and people working or living adjacent to where the colony was located, most did (Table 6A). The most common method utilized was to meet one-on-one to explain the program and educate complainants. For example, regarding issues with neighbors, 73% met one-on-one to explain the program. Others dropped educational flyers into letterboxes or provided cat deterrents, while a few removed the cats to foster care for protection. One respondent said they held a community meeting for neighbors, and other respondent held one for people working adjacent to the colony.

**Table 6A:** When TNR commenced, what did you or your organization do to resolve the complaints or issues?

	**Neighbors respondents (*n* = 15)**	**People working adjacent (*n* = 6)**	**People living (*n* = 16)**
No attempts were made to resolve	1 (7%)	0 (0%)	2 (13%)
Met one-to-one to explain the program and educate	11 (73%)	2 (33%)	10 (63%)
Spoke one-to-one by phone to explain, program, and educate	1 (7%)	0 (0%)	1 (6%)
Brought cats into foster care to protect them	3 (20%)	1 (17%)	3 (19%)
Dropped flyers in letterboxes to explain the program and educate	2 (13%)	0 (0%)	1 (6%)
Held a community meeting	1 (7%)	1 (17%)	0 (0%)
Provided cat deterrents for their use	2 (13%)	0 (0%)	1 (6%)
Other free-form comments	6 (40%)	2 (33%)	4 (25%)

In free-form comments, respondents said to resolve issues or conflict, one strategy they used was to educate the community using information flyers distributed within the community and personalized notices on community noticeboards, with text in multiple languages (Table 6B). Others addressed nuisance issues by relocating feeding areas and addressing parasite control concerns.

**Table 6B:** When TNR commenced, what did you or your organization do to resolve the complaints or issues?

**COMMUNITY EDUCATION ABOUT TNR**
“I placed information on a community noticeboard in the building hallway, with a picture of the cats and their individual story in English, Korean, Chinese and Spanish.” “We advised that desexing the colony was a first step to stabilize numbers while removing kittens. Next was to remove friendly cats for rehoming. Third was to chip and support remaining cats which could not be adopted due to being unfriendly.” “Sometimes I was laughed at, or told off when people were not aware. I would take my time, explain what I could and turn around their attitudes, where possible to be supportive, or at worst, uninterested in my activities.”
**RELOCATION OF FEEDING SITE FOR COLONY**
“I have helped out with other colonies where people were abused and threatened, and I showed the volunteers how to relocate a feeding station, by short distances over time, to a more supportive, less abusive location.” “If you scout out an area, you can usually find somewhere the cats will happily go to receive their regular food. Use visual and verbal cues to find the new locations and move with them – it is an effective methodology. Cats are intelligent and opportunistic, and will go where it works for them and you, when worked with properly.”
**ADDRESSING PERCEIVED CONCERNS ABOUT HEALTH RISK**
“I also parasite treated areas with diatomaceous earth (food grade), so that there were no potential complaints about bugs. I previously ‘flea treated’ sleeping patches in gardens, under shrubs, in sleeping boxes with ‘Frontline spray’—we never had flea issues. Mosquitoes used to feast on me in the early days until I fixed up the area with clean, regularly refreshed water bowls, and got rid of old pots and containers from the hoarder's place—access was all I needed and then it was fixed quickly!”

Respondents were asked how successful they thought the outcomes of their attempts to resolve the issues with neighbors and people who lived where the colony was located. For example, for conflicts with neighbors, 42% of respondents believed their attempts to resolve the complaint were somewhat or very successful, but 57% felt they were somewhat or very unsuccessful resolving disputes with those working near the colonies (Table 7). One respondent reported that the “main complaints came from anti-cat members of the university community. We did not approach them directly—actions (and results) spoke louder than words.”

**Table 7:** What were the outcome of these attempts to resolve these complaints or issues?

	**Neighbors (*n* = 14)**	**People working adjacent (*n* = 7)**	**People living (*n* = 12)**
Very unsuccessful	4 (29%)	3 (43%)	3 (25%)
Somewhat unsuccessful	3 (21%)	1 (14%)	2 (17%)
Neither successful nor unsuccessful	1 (7%)	1 (14%)	1 (8%)
Somewhat successful	3 (21%)	1 (14%)	4 (33%)
Very successful	3 (21%)	1 (14%)	2 (17%)

In free-form comments, respondents gave examples where complaint resolution was unsuccessful, despite attempts to explain TNR. For example, “when I spoke to neighbors and advised the cats were desexed, microchipped and vaccinated, it was rejected and they treated me as a liar.” For some respondents, lack of stakeholder supported resulted in them stopping TNR at that site. “Council rangers were called by an aggressive resident. The ranger was sarcastic and dismissive because the cats were not chipped to the resident feeding them. We tried to explain she was elderly and had dementia, and that this was the first step before continuing to remove suitable cats. We stopped due to fear of reprisal, and the cats have continued to breed,” without council involvement and “some residents wouldn't allow us to return the cats or were hostile to the cats. As a result, they were brought into foster care, regardless of whether they were socialized or not, and we stopped TNR at that site.”

### Current Perceptions of Various Stakeholders to TNR

#### Perceptions of Current Awareness and Attitudes of the Agency Legally Responsible for Investigating Animal Cruelty

Respondents were asked which agency they believed was legally responsible for investigating animal cruelty in their state and most (68%, 15/22) believed it was the RSPCA, while 18% (4/22) indicted they thought it was the local government agency (council). The most common response was that the agency responsible for animal cruelty was not aware of TNR occurring at that location (41%) and 22% believed the agency were aware, but did not acknowledge their awareness (Table 8).

**Table 8:** Perceived current awareness and attitudes to TNR by authorities responsible for animal welfare, landowners, neighbors, workers, and residents living where this colony was located.

**Reasons**	**Agency legally responsible for investigating animal cruelty (%), (*n* = 27)**	**Landowners or authorities responsible for land (%), (*n* = 26)**	**Neighbors (%), (*n* = 28)**	**Workers (%), (*n* = 21)**	**Residents (%), (*n* = 27)**
Threatened to take legal action	2 (7%)	2 (8%)	2 (7%)	2 (10%)	1 (4%)
Very antagonistic but took no direct action to prevent occurrence	3 (11%)	3 (12%)	2 (7%)	0	2 (7%)
Tension Only	0	1 (4%)	1 (4%)	1 (5%)	2 (7%)
I do not think they were aware of it occurring	11 (41%)	5 (19%)	4 (14%)	5 (24%)	3 (11%)
Stakeholder/s were aware of it but did not acknowledge they were aware of it occurring	6 (22%)	5 (19%)	5 (18%)	0	4 (15%)
Stakeholder/s supported it but did not provide assistance with resources (in-kind or cash)	1 (4%)	3 (12%)	8 (29%)	3 (14%)	5 (19%)
Stakeholder/s supported it and provided assistance with access to resources (e.g., human resources, desexing vouchers or assistance with desexing, traps, other assistance or cash)	0	2 (8%)	3 (11%)	1 (5%)	5 (19%)
Don't know	2 (7%)	2 (8%)	1 (4%)	4 (19%)	1 (4%)
Not applicable	2 (7%)	3 (12%)	2 (7%)	5 (24%)	4 (15)

*Respondents could select more than one response (n = respondents for each question)*.

Two (7%) respondents reported they were threatened with legal action in Brisbane, Queensland; one indicated a summons for feeding non-domestic cats was issued, and another volunteer was fined for feeding “non-domestic” cats and for removing two kittens for adoption. In free-form comments, one respondent reported that a NSW colony managed by a small welfare group was privately supported by the RSPCA, because of unclear legal interpretation of the law relating to unowned community cats. Another NSW respondent indicated that their local veterinarian would destroy surrendered stray cats. In NSW, Western Australia (WA) and Victoria, respondents reported the RSPCA were either not supportive of TNR, unwilling to assist with cat management, or unable to support TNR due to current shelter overcrowding. One respondent said the “RSPCA offered to kill them for a fee, if we trapped them and took them in.”

#### Perceptions of Current Awareness and Attitudes of Those Stakeholders in the Vicinity of the Colony (Landowners, Neighbors, Workers, and Residents)

More landholders were aware of TNR activities currently, compared with when the colony commenced (16 compared to 10). Support was more polarized with more reporting negative attitudes including threatening to take legal action (2 vs. 1), and more reporting supportive attitudes including providing assistance with access to resources (1 vs. 2) (Tables 3, 8).

The most common response regarding current behavior of neighbors and residents was that authorities supported it, but did not provide assistance with resources, either in-kind or cash. However, some reported negative feedback ranging from either tension or antagonism but no direct action, to neighbors and residents threatening to take legal action. In contrast, the most common response (24%) regarding behavior of workers was that respondents did not think workers were aware TNR was occurring (Table 8).

### Most Influential Stakeholders Currently and Why They Supported TNR

In free-form comments, 21 respondents stated that stakeholders who supported TNR included residents and tolerant or supportive neighbors (10, 48%), cat rescue groups (*n* = 4), cat welfare agencies (*n* = 2), other private supportive individuals and friends (*n* = 2), municipal council staff (*n* = 1), university staff (*n* = 1) and supportive veterinarians (*n* = 1). Seven said that they were the most influential stakeholder supporting TNR, either alone (*n* = 5), or with help from friends (*n* = 2).

Free-form comments stated several council offices became tolerant of TNR after receiving qualified information on TNR. “Council's ‘solution’ to feral cat problems was to issue fines to anyone feeding them. I told them that we were desexing the cats, and rehoming where possible. They were fine with this,” or “they just didn't care, the council companion animal officer said it was on private land so it was not their problem.” Other stakeholders accepted the cats over time if the issues resolved. “Most people have either grown fond of the remaining cats, or are benign and don't care, because it is managed and not a problem.”

When respondents were asked their opinion on stakeholder's main reasons for supporting TNR, the most common beliefs were that TNR was a humane approach to managing community cats (86%), to improve the welfare of cats and kittens (86%), and they believed TNR is an effective strategy to reduce the community cat population over time (83%) (Table 9). A few nominated reasons associated with cat-related complaints such as noise, smell or wildlife predation. Another respondent indicated stakeholder support was likely due to cats providing rodent control, and commented TNR was supported “to stabilize the colony without removing it, because it is managing a massive rodent issue.”

**Table 9:** Thinking about those who support TNR occurring at this colony, what are their main reasons for this? Respondents could select more than one reason.

**Reasons**	**Number and % of respondents nominating reason why stakeholders supported TNR occurring with this colony (%), (*n* = 29)**
Because it is a humane approach to managing community cats	25 (86%)
To improve the welfare of the cats/kittens	25 (86%)
Because it is an effective strategy to reduce the community cat population over time	24 (83%)
To improve the health of the cats/kittens	21 (72%)
To resolve complaints related to smell from cats	6 (21%)
To resolve complaints related to wildlife injury/deaths	6 (21%)
To resolve complaints related to noise from cats	5 (17%)
Because an organization was funded to do a TNR program	1 (3%)

The most common method of supporting TNR work was by providing cash or food (5/11, 45%), often through fundraising efforts, and one assisted with adoptions. Some stakeholders then became involved in TNR themselves (2/11, 18%). Another commenced community relationship development and provided ongoing education to other stakeholders. One supportive veterinarian provided pro-bono desexing. Another respondent stated that their veterinarian introduced them to a cat adoption center and expounded the merits of TNR to the council. As a result, the council provided workers to remove kittens.

### Organizational Support to the Community for TNR

Of the 30 respondents to the opinions section of the questionnaire, 36% conducted TNR as part of an organization. If the organization provided support for the community to do TNR, the most common support provided was discounted or free desexing (93%), traps (86%), and advice on TNR (79%) (Table 10). Training sessions on TNR were less commonly offered (21%).

**Table 10:** For respondents involved in TNR as part of an organization, support provided by the organization to the community to undertake TNR activities (*n* = 14)*.

**Support provided**	**Number and % of respondents who indicated the type of suppprt their organization provides to the community to undertake TNR**
Advice on TNR	11 (79%)
Training sessions on TNR	3 (21%)
Traps	12 (86%)
Food	8 (57%)
Discounted/ free desexing	13 (93%)
Discounted/ health care for cats/kittens	8 (57%)

* *Although only 10 respondents to question 1 of the survey indicated they were part of an organization, 14 answered this question later in the survey. It is unknown if the additional four were loosely connected with an organization and therefore chose to answer the question, or if they misread the question. We have included the answers from all 14 respondents*.

### Main Challenges

In free-form comments, the major challenges nominated by 15 respondents were uncooperative stakeholders (47%, *n* = 7). Respondents commented that one of the major challenges was from unsupportive stakeholders who did not like cats or animals (37%) and had negative opinions toward cats. Others mentioned difficulty catching poorly socialized cats (29%), lack of financial support (27%) and unorganized feeding, leading to multiple sources of food for cats, making trapping more difficult (20%). One respondent indicated that a veterinary clinic preferred euthanasia rather than trying TNR. Other challenges cited were ambiguous legislation or perceptions that culling was successful for population control (*n* = 1), threats to native wildlife (*n* = 3), or the perception that TNR would increase the cat population (*n* = 1).

### The Main Reasons for Those Who Do Not Support TNR Occurring at This Colony

Respondents were asked what they believed were the three main reasons for those who did not support TNR occurring at this colony. The main reasons cited were related to not liking cats or animals (58% of respondents), ignorance (37%) and concerns about wildlife predation (37%) (Table 11A).

**Table 11A:** Thinking about those who do not support TNR occurring at this colony, what are their three main reasons for this? (Respondents = 19).

**Theme**	**Number and % of respondents nominating reason why TNR was not supported**
Don't like cats/animals	11 (58%)
Ignorance	7 (37%)
Wildlife predation	7 (37%)
Legislation regarding unowned cats	4 (21%)
Support culling	2 (10%)
Apathy	2 (10%)
Fear that it will lead to too many/more cats	2 (10%)
Welfare concerns	2 (10%)
Nuisance	2 (10%)
Spread disease to people	1 (5%)

In free-form comments about reasons those who did not support TNR occurring at this colony, respondents who cited a belief that it was ignorance, or a lack of interest in humane control options also suggested these stakeholders attitudes were influenced by a personal dislike of cats. Respondents cited council by-laws as a deterrent to support for TNR (Table 11B).

**Table 11B:** Opinions on the lack of support toward TNR.

**IGNORANCE**
“Some people think they should all have homes found, but they don't understand that these cats are feral and hard to home.” “Non-supporters think it is more humane to just put them all down if they can't be rehomed.”
**LACK OF INTEREST**
“They have no interest in it. I believe they would think it unnecessary.”
**ISSUES WITH COUNCIL BY-LAWS**
“Brisbane City Council cat and kitten trapping program will never support TNR due to stating cats and kittens that are trapped are all feral, and that it is illegal to do TNR, or to rehabilitate and rehome them.” “TNR is actively discouraged by council rangers. They imply illegality without providing more details to the home owner.”
**DISLIKE OF CATS**
“They don't want cats around” “Most of them want the cats gone. Poof!”

## Discussion

This study of perceived stakeholder support and opposition for “grass-roots” TNR activities in urban Australia provides an insight to the challenges faced by respondents conducting TNR. Key findings of our study were that the main reasons respondents initiated TNR was because they believed it was a humane and effective way to manage community cats. They frequently encountered issues with authorities, landowners, neighbors, and people living and working in the area. Nearly all took action to resolve these issues, although resolution was not always successful. This study highlights the difficulty for individuals and community cat welfare organizations involved in TNR to manage urban cat colonies when it is not supported by government by-laws or major welfare agencies such as the RSPCA. These findings support the need for legislative change to facilitate best-practice TNR.

### Demographics of Respondents

The 30 respondents in this study were surveyed about their perceptions of awareness and attitudes of stakeholders to their TNR activities. Stakeholders, defined as a person, group or organization that had an active interest in the TNR activity, typically represented organizations involved with compliance to relevant legislation, or were individuals in the vicinity of the colony being managed. The demographics of the respondents to questions on stakeholder support reflected the demographics of the entire cohort, on whom we have reported various aspects of their TNR activities and effect on colony size. Respondents were predominately female and 39% were aged 46–55 years of age (2). The gender bias toward females aligns with reports of more caring attitudes toward animals and more frequent involvement in feeding stray cats (40–42). However, selection bias may have skewed these results; and a recent Australian study found a similar proportion of males and females fed urban stray cats (43). Further research that minimizes selection bias is required to clarify the gender ratios of cat feeders in different geographical locations.

### Reasons Why Respondents Began TNR for That Colony

The main reasons cited by respondents' for beginning TNR was that it was an effective way to reduce the community cat population over time and a humane approach to cat management. Overseas research consistently shows that, provided best practice is implemented, TNR reduces cat numbers in colonies over time (13, 18, 21, 44–47). Two studies in Florida have separately demonstrated significant reductions in cat populations when TNR was initiated (18, 40). Of particular interest is a larger study that used shelter cat intake as a surrogate measure of free roaming cats in a whole zip code in Florida (21). When cats were desexed at a rate of 60 per 1,000 residents, shelter intake dropped from 13 to 4 cats per 1,000 residents and was 66% lower after 2 years. In contrast, in the control area where an average of 8 cats were desexed per 1,000 residents, shelter intake decreased only 12%, and shelter euthanasia and intake rates were 17.5 and 3.5-fold higher, respectively, compared to the treatment zip code. Reduction in cat colony size is also demonstrated in Australian populations. For example, a 31% decrease in colony size occurred over 2 years in colonies managed by respondents to this stakeholder attitudes survey, and a reduction from 69 to 15 cats occurred, despite influx of immigrant cats in a program run by the University of NSW (8).

Other major reasons for involvement with TNR were concerns for cat welfare and to improve cat health within the colony. Similar reasons were cited in Florida; the most cited motivations were sympathy and ethical concerns to care for hungry, injured or unowned cats, and most did not want cats killed (40). A recent study in Brisbane, Australia, found that 79% of residents preferred TNR to lethal methods (43).

Authorities and policy makers often cite concern for cat welfare as a reason not to support TNR. For example, the Australian Veterinary Association do not support TNR, citing poor welfare once released. This belief is not supported by evidence. Similar levels of welfare and life expectancy have been reported between pet and colony cats in Australia and New Zealand (8, 34). In the USA, unowned cats had similar rates of infectious diseases as owned cats (8, 40, 44, 46, 48, 49). One Australian study found that the prevalence of feline immunodeficiency virus (FIV) was lower in shelter cats than owned cats with outdoor access (50).

### Awareness by Authorities at Commencement of TNR and Permissions Sought

In Australia, state government laws pertaining to biosecurity and domestic animal management are typically administered by councils (local government area similar to counties in USA). Councils also have their own by-laws relating to registration (licensing), microchipping, and cat containment. In addition, the RSPCA is responsible for animal welfare in most states. At the commencement of TNR, nearly half of the respondents believed the authorities were not aware of their work in the colony. Some believed the authorities were aware but did not prevent it. One respondent was threatened with legal action. More than half the respondents sought permission, mainly from property owners and councils, and none requested permission from state government authorities or RSPCA. Those that requested permission received it, although only one was given proof in writing.

In some Australian states, the term “domestic cats” includes urban strays with no identified owner. For example in Tasmania, residents are prohibited from abandoning cats under animal welfare legislation. Respondents in our study clearly were not “abandoning” cats; cats were fed daily, provided routine healthcare (such as antihelmintics and vaccinations), and most kittens were vaccinated, and treated for fleas and intestinal parasites (2). However, 18% of respondents reported that the agency they thought responsible for investigating animal cruelty was very antagonistic and some respondents reported they threatened legal action.

To decrease cat intake and euthanasia in shelters and pounds, local government and major welfare agencies typically aim to educate cat owners about the importance of desexing. This focus on cat owners ignores research showing that most cats entering shelters and pounds are semi-owned or stray. In Brisbane it has been demonstrated that 15% of respondents had fed stray cats, and 9% of respondents in an internet survey had fed them daily. Hence, semi-ownership is common and most respondents of the Brisbane study disagreed with legislation that prohibited stray cats being fed without a permit (42, 43). Authorities need to focus on semi-owners and stray cats, and should be aware and supportive of managed TNR programs. Evidence clearly demonstrates the long-term success of TNR in reducing cat numbers (13) and that TNR has greater community support than culling (43, 51–53).

### Perceptions of Awareness and Behavior of Various Stakeholders at Commencement and at the Time of Reporting

Only one third of respondents perceived that stakeholders supported TNR occurring at the site. These stakeholders represented those legally responsible for the land where the colony was located or individuals living or working in the vicinity. Some reported negative attitudes, ranging from tension to threatening to take legal action. Complaints were mostly from neighbors (68% of respondents), and some were from people working adjacent to the colony (33%). This may have reflected colony location; most were at private residences. At the time of reporting, support was more polarized than at commencement, as more stakeholders became aware of TNR in the area.

Previously, instances of conflict between cat caregivers and property owners have been reported by 86% of welfare workers involved with TNR (54). Conflict can discourage volunteers from working with welfare organizations (54). In our study, respondents reported removing cats to foster homes and stopping TNR after residents were antagonistic.

An Australian study demonstrated that support for non-lethal management involving desexing was generally greater amongst those who owned pet cats, fed stray cats or were female and younger. In contrast, those who did not own pet cats, were aware of stray cats in their vicinity but did not feed them, or were male and older were more likely to choose lethal management. The only significant predictor of choice of lethal management was a belief that cats spread disease to humans (43). This was also mentioned by one of our respondents as a main reason for lack of stakeholder support.

#### Public Health Concerns

Urban stray cats are often portrayed to be disease ridden, however a study of 553 cats in Northern Florida found them to be of no greater disease threat to humans or pets than pet cats (49). The National Association of State Public Health Veterinarians (NASPHV) state there are potential risks to human public health by zoonotic diseases including rabies, bartonellosis and toxoplasmosis (55). Rabies is the most serious of these diseases and does not occur in Australia. In USA, when comparing unowned to owned cats, there are similar or lower prevalence rates of many potentially fatal feline diseases and diseases of concern to humans, such as *Toxoplasma gondii* and *Bartonella henselae* (cat scratch fever) (49). Many of the diseases of concern to humans are transmitted via bites or fleas, and for these diseases, transmission from pet cats is more likely than from stray cats, where close contact with humans is less likely (56, 57).

Australia has some of the highest infection rates for *Toxoplasma gondii* in cats in the world (58). After infection, cats shed oocysts (eggs) for 2-to-3 weeks, after which they acquire immunity (59). TNR programs likely reduce environmental contamination with toxoplasma oocysts more than trap and kill programs because mature cats are desexed and returned to their home location or colony. Their mature age means they are more likely to have previously been infected, and are immune to toxoplasmosis (60, 61). Additionally, if cats older than 1 year become infected, they shed fewer oocysts than younger cats (62). In contrast, in trap and kill programs, young immunologically naïve kittens are continuously being born, become infected and shed *Toxoplasma* oocysts.

### Actions by Respondents to Resolve Complaints and Issues

Of respondents reporting issues or complaints, over 90% took action to try to resolve the issues, most commonly meeting one to one to explain the program and educate. Other actions included letterbox flyers to explain the program, community meetings or providing cat deterrents. One respondent detailed how she personalized the individual cats in the colony with pictures and their stories in multiple languages on the community notice board. A proactive response to stakeholders' concerns is recommended by the ASPCA in their handbook on TNR (63). Conflict over cat management can arise from preconceptions, and clashes of goals, values and beliefs, which are in-turn influenced by gender, age, and level of education of the parties (54). Gaining trust, avoiding conflict, and listening to alternative opinions are suggested as a strategy for caregivers (64). The success of these types of approaches is supported by a study with respondents from 24 states in the USA. Dialogue and debate, along with empirical evidence, were necessary for identifying common goals on animal management and welfare (41).

### Influential Stakeholders Who Facilitated TNR Initially at the Colony

The most influential stakeholders who facilitated TNR were from community cat welfare agencies and rescue groups, municipal staff, veterinarians, tolerant neighbors, and supportive members of the public. One respondent indicated that she only enlisted assistance from close friends due to concerns about the illegality of TNR. Some influential stakeholders provided education on trapping, and some provided financial assistance for desexing and medical care. Long-term success of a control program depends on financial support, individual commitment and public support. It requires the interaction between informed individuals working in the field and the authorities who can make supportive decisions on humane stray cat management based on scientific evidence.

### Reasons for Stakeholder Support

The main reasons perceived for stakeholder support were population reduction and improving cat welfare and health, which were similar to the reasons given by our respondents, and those cited in the literature (51). In an Irish study, stakeholders demonstrated a positive attitude toward cat management if they had made an informed decision that TNR was an effective way to control cat populations (65).

Economic benefits also result from population reduction via TNR. In Santa Clara County, shelter costs for low-cost spay and neuter programs were ~$23.21 per cat compared to husbandry costs for the average litter size of 3.5 kittens approaching $900 (51, 66). Effective lethal control requires killing 15-to-20 times more cats than current rates in Australia and is prohibitively expensive for municipalities with limited budgets (7, 67, 68). Despite a number of reports in the literature of effective large-scale TNR programs, there are none from Western countries of effective trap and kill programs (13–15, 21).

### Support Provided by Organizations to the Community to Undertake TNR

Respondents participating in TNR as part of an organization indicated that support was provided through discounted or free desexing, provision of traps and advice on TNR. Stakeholders participated themselves or provided dialogue to municipal authorities or welfare organizations. The resources of large influential stakeholders were not used to support TNR, because it is illegal in most parts of Australia. In contrast, in USA there is support for TNR from most of the major welfare organizations, including Humane Society USA (HSUS), American Society for Prevention of Cruelty to Animals (ASPCA), Society for Prevention of Cruelty to Animals (SPCA) and Best Friends. In addition, foundations with substantial financial resources such as the Maddie's Fund and PetSmart Charities, contribute grants of up to $500,000 for TNR (69, 70).

### Main Challenges

Lack of resources was an impediment, but if TNR were legalized, this could be addressed by recruiting community members who are already feeding unowned cats to participate in resourcing. Municipalities, welfare agencies and individuals could support items which have a direct cost, such as the provision of traps and funding for desexing.

Some respondents reported difficulty catching cats, as catching poorly socialized cats frequently takes more time than anticipated, with an average of 6 days to trap the whole colony reported in one study from USA (46). Multiple food sources compounded this issue.

Uncooperative stakeholders was another significant challenge, and respondents cited ambiguous legislation and perceptions that TNR would increase cat populations as impediments. Legislation pertaining to abandonment of cats and feral pests must be amended in Australia. This would allow major animal welfare organizations with substantial resources such as the RSPCA to recruit caretakers without threat of litigation. These organizations are less likely to be confronted by unsupportive stakeholders because their principles are supported by peer-reviewed literature, and they are potentially better placed to develop and maintain community relations. Furthermore, they would benefit substantially by reducing cat intake into their shelters. They can also better leverage economies of scale to reduce desexing costs by undertaking large-scale desexing programs, possibly in conjunction with universities training veterinary students (71). Both in Australia and overseas, successful collaborations between cat welfare groups and universities with veterinary schools have occurred (17, 72). Amending legislation obstructive to TNR and implementing best practice, will minimize the risk of complaints and help protect the cats.

### Limitations

The biggest limitation of this study is the small data set. The length of the survey, taking ~1 h to complete, meant that only 53% of respondents completed questions pertaining to this second part of the study. This study also highlights the difficulty in collecting data on TNR activities when TNR is not supported by government laws or major welfare agencies such as the RSPCA. One of the obstacles to research on TNR in Australia is the concern of potential respondents to the consequences of detection by municipal councils or animal welfare organizations legally responsible for animal welfare, which may deter participation. We used Maggie's Rescue to collect the data because of concerns that participants could be traced if data were collected directly by the university, which is government funded and subject to Freedom of Information legislation. Our approach to collecting data via snowball sampling makes it difficult to determine sampling error or deduce inferences about the entire population of people involved in TNR activities across Australia. This method also has the potential for bias because respondents were self-selected and were generally computer and Internet literate with readily available access to the Internet.

## Conclusions

This paper highlights the challenges faced by volunteer careers in the urban setting. It is the first study to report stakeholder support for “grass-roots” TNR activities in urban Australia. Comments by respondents provide a rich insight to the diverse challenges faced when conducting TRN in urban environments in Australia. TNR and caring for unowned cats in the urban environment is illegal in most jurisdictions in Australia. Respondents worked alone or with trusted friends, self-funding their activities in often difficult situations. They initiated TNR at those colonies because they believed it was a humane and effective way to manage community cats. Respondents frequently encountered complaints from authorities, landowners, neighbors, and people living and working in the area. Nearly all took action to resolve these issues, although their actions were only partially successful. Two respondents were threatened by legal action, and although no respondents were fined for releasing or “abandoning” cats, respondents reported that volunteers were fined for feeding or removing kittens for adoption in Brisbane Queensland, which has some of the most restrictive legislation of all Australian states.

Based on the previously reported effectiveness of these respondents in decreasing colony size through TNR (2), and similar reports in the literature from Australia and internationally, it is recommended that TNR be legalized in Australia in urban and periurban areas. This may reduce antagonism with stakeholders, and also facilitate greater resources being made available for desexing, feeding, and health care by large welfare agencies, so best practice can be undertaken, including that for dispute resolution.

## Author Contributions

JR: conceived the study. JR and KT: designed the questionnaire. JR and AH: analyzed the data and wrote the paper with input from KT.

### Conflict of Interest Statement

The authors declare that the research was conducted in the absence of any commercial or financial relationships that could be construed as a potential conflict of interest.
